# Unhealthy dietary patterns among healthcare professionals and students in Mexico

**DOI:** 10.1186/s12889-018-6153-7

**Published:** 2018-11-09

**Authors:** Alejandra Betancourt-Nuñez, Fabiola Márquez-Sandoval, Laura I. González-Zapata, Nancy Babio, Barbara Vizmanos

**Affiliations:** 1 Cuerpo Académico UDG-454, Alimentación y Nutrición en el proceso Salud Enfermedad. Departamento de Disciplinas Filosófico, Metodológico e Instrumentales, Centro Universitario de Ciencias de la Salud, Universidad de Guadalajara, Sierra Mojada # 950, CP, 44340 Guadalajara, Jalisco Mexico; 2 Doctorado en Ciencias de la Nutrición Traslacional. Cuerpo Académico UDG-454, Alimentación y Nutrición en el proceso Salud Enfermedad. Departamento de Reproducción Humana, Crecimiento y Desarrollo Infantil, Centro Universitario de Ciencias de la Salud, Universidad de Guadalajara, Sierra Mojada # 950, CP, 44340 Guadalajara, Jalisco Mexico; 3 Grupo de Investigación en Determinantes Sociales del Estado de la Salud y la Nutrición, Escuela de Nutrición y Dietética, Universidad de Antioquia, Medellín, Antioquia Colombia; 4 Human Nutrition Unit. Department of Biochemistry and Biotechnology, Rovira i Virgili University, Sant Joan de Reus Hospital. IISPV, Reus, Spain; 5 CIBER de Fisiopatología de la Obesidad y la Nutrición (CIBEROBN), Instituto de Salud Carlos III (ISCIII), Madrid, Spain

**Keywords:** Dietary pattern, Health personnel, Mexico, Young

## Abstract

**Background:**

While dietary patterns (DPs) enable the combination of foods that make up a person’s habitual diet to be known, little is known about the DPs of health sector professionals. The objective of this study was to describe the DPs of healthcare students and professionals and assess their association with sociodemographic, lifestyle, anthropometric and biochemical characteristics.

**Methods:**

Cross-sectional design. A sample (*n* = 319) of healthcare students and professionals in apparent good health who studied or worked at the University of Guadalajara (Mexico) was selected. A semiquantitative food intake frequency questionnaire validated on a Mexican population was administered. Questions covering sociodemographic factors, smoking habits and physical activity were asked. Weight, height, waist circumference, blood pressure, triglycerides, glucose, HDL-cholesterol, LDL-cholesterol and total cholesterol were also measured. DPs were generated from a principal components analysis of 25 food groups, and associations were analyzed using logistic regression adjusted for age and sex.

**Results:**

The majority of participants were younger than 29 years (84%), women (71.2%) and students (59.6%). Three DPs were identified: “Traditional Westernized”, “Healthy” and “Animal protein and alcoholic beverages”. After adjustment, the “Traditional Westernized” DP was positively associated with being younger than 22 years (OR: 2.15; 95%CI: 1.1–4.1); the “Healthy” DP was positively associated with having a daily energy expenditure from physical activity greater than 605 kcal (OR: 4.19; 95%CI: 2.3–7.5), and it was negatively associated with being younger than 22 years (OR: 0.48; 95%CI: 0.2–0.9); and the “Animal protein and alcoholic beverages” DP was positively associated with being male (OR: 3.07; 95%CI: 1.8–5.1) and a smoker (OR: 2.77; 95%CI: 1.2–6.3). No association was found between DPs and anthropometric and biochemical characteristics.

**Conclusions:**

Among the participants evaluated, healthy DP was associated with being physically active while unhealthy DPs were associated with being younger than 22 years, male and a smoker. These data suggest that being knowledgeable about health does not ensure that individuals will engage in healthy behaviors. As is the case among the general population, training and individual efforts aimed at achieving healthy behaviors must be reinforced by initiatives undertaken by social groups, social institutions, the community at large as well as political and business leaders.

## Background

In Mexico, as is the case in other Latin American countries, nutritional patterns are undergoing a transition [1, 2]. The Mexican diet is shifting towards higher intake levels of foods high in saturated fat, refined carbohydrates and sodium [1–3]. More than 50% of Mexico’s population have insufficient levels of fiber intake and exceed the recommended saturated fat and added sugar intake limits (10% of total energy intake each) set by the World Health Organization [3]. In addition, more than 50% of this population group exceed the recommended intake limits for processed meats, sugar sweetened beverages (which are the main source of added sugars in the diet and account for 9.8% of total energy intake) and foods high in saturated fat and/or added sugar, consisting of mostly processed and packaged foods such as snacks, pastries, desserts, and confectionary foods whose saturated fat and/or added sugar content is > 13% and which comprise 16% of total energy intake [3]. Furthermore, only 1–23% of Mexicans adhere to intake recommendations for legumes, seafood, fruit and vegetables, and dairy products [3]. Consequently, a high proportion of Mexican adults (≥20 years of age) do not meet minimum nutrient requirements [3]. These dietary trends, coupled with lower physical activity levels, have resulted in higher prevalence rates of obesity, type 2 diabetes mellitus and hypertension, conditions which are among Mexico’s most serious public health problems [1].

The amount of detailed data available on the eating habits of health sector professionals is very limited, especially in Mexico. Most studies only assess the intake of certain food groups or nutrients by this population group. However, these data show that – as is the case among the general population – most healthcare students and professionals from countries such as Mexico [4], Puerto Rico [5], Brazil [6], Colombia [7], the United Kingdom [8, 9], the United States [10–12] and Australia [13] do not follow their countries’ dietary recommendations. Most of them consume less than the recommended daily amounts of fruits and vegetables [4–13], whole grains [4, 5], dairy products [5], and protein [5]; less than a half drink eight or more glasses of water daily [5, 8, 9]; more than 30% consume foods high in fat and sugar on a daily basis [8, 9]; more than a half (62.1%) have inadequate dietary patterns (< 50% meet dietary recommendations) [5]; and most of them (74.5%) have a diet that could be classified as “unhealthy” [4].

One way for health professionals to optimize their role in promoting the health of the general population is to follow a healthy lifestyle themselves. It has been shown that health professionals who have healthy eating habits often talk to their patients about these habits [7, 10, 14]. For example, in a sample of medical students [7, 10] and professionals [14] from the United States [10, 14] and Colombia [7], a positive association was found between fruit and vegetable intake and favorable attitudes toward nutrition counseling [7, 10, 14]. In addition, the health-related behaviors of physicians seem to influence the attitudes and motivation of their patients with regard to making lifestyle changes [15]. It is therefore of prime importance for health professionals to engage in healthy behaviors such as following a healthy diet, not only for the sake of maintaining a low cardiometabolic risk status but also as a way to promote healthy behaviors through their professional work.

Because of the influence that the health-related behaviors of health professionals may have on the behaviors of the general population, studies are needed to carefully evaluate and monitor the nutritional habits of these professionals. One alternative and complementary approach to the assessment of food or nutrients intake is to obtain a description of dietary patterns (DPs) [16] because they reflect how different foods and nutrients are combined in a person’s habitual diet [16, 17]. Describing DPs is of interest and is more realistic than other techniques because people routinely consume food in particular combinations [16, 17]. A variety of DPs have been described in scientific literature, and several of them are similar across populations [18]. However, to our knowledge very little or nothing has been published regarding the DPs of healthcare professionals and students. It is important to describe DPs in diverse population groups, such as healthcare students and professionals in Mexico, given that the constituent foods of DPs may vary according to socioeconomic status, culture, ethnic group, sex, food preferences and food availability [16], among other variables. That is, different DPs may exist within different populations, and individual DPs may also undergo changes over time [16].

It is also important to know whether particular sociodemographic, lifestyle, anthropometric or biochemical characteristics may favor adherence to these DPs. This dietary information will help to detect any problem areas and, where appropriate, suggest specific actions to promote the selection and combination of those foods that will directly benefit the health of this population group and thus indirectly benefit the health of the general population.

The objective of this study was to describe the DPs in a sample of healthcare professionals and students from the University of Guadalajara (Mexico) and to evaluate their association with sociodemographic, lifestyle, anthropometric and biochemical characteristics.

## Methods

### Study sample

This cross-sectional study included participants from Mexico. It is derived from the multicenter study entitled Latin America Metabolic Syndrome (LATINMETS), which includes participants from five Latin American cities: Guadalajara (Mexico), Medellín (Colombia), Viçosa (Brazil), Buenos Aires (Argentina) and Asunción (Paraguay). The research teams in these cities together form the Ibero-American Network for the Study of Metabolic Syndrome (RIBESMET), which is coordinated by Rovira i Virgili University in Reus, Spain.

An open invitation to participate voluntarily in this study was extended to professionals (university graduates) and university students (in their final semesters) who were: over 18 years of age; primarily involved in the health-related fields of nutrition, psychology, dentistry, nursing, medicine or pharmaceutical biochemistry; and working or studying at the University of Guadalajara or the Civil Hospital of Guadalajara in the period from January 2011 to July 2013. We did not include pregnant or lactating women, people taking corticosteroids or oncological patients.

### Sociodemographic and lifestyle variables

Data for some sociodemographic characteristics such as age, sex, occupational status (student or professional) and health field were collected through interviews. Lifestyle characteristics included smoking status (non-smoker or smoker) and physical activity (PA), the latter of which was assessed using a validated Spanish-language version of the Minnesota Leisure-Time Physical Activity Questionnaire [19]. The amount of time spent weekly on PA and daily energy expenditure (kilocalories/day) from PA (MET*minutes*days per week) were calculated based on the frequency of each activity and the average time spent per day. For the purposes of interpretation, daily energy expenditure was categorized into tertiles, and weekly time spent on PA was categorized according to World Health Organization recommendations (≥150 min per week o < 150 min per week).

### Anthropometric variables

The study’s researchers measured body weight (using a TANITA UM-06® electronic scale, accurate to 0.1 kg), height (using a SECA® stadiometer, accurate to 0.1 cm) and waist circumference (fiberglass measuring tape, 0.1 cm) according to International Society for the Advancement of Kinanthropometry standards [20]. Body Mass Index (BMI) was calculated [weight (kilograms)/ height^2^ (meter)] and each subject was classified according to World Health Organization criteria [21]. In addition, waist circumference was measured and used to classify subjects as either having abdominal obesity (≥80 cm in women and ≥ 90 cm in men; criteria for Central and South American populations) [22] or not having any health risk based on this factor.

### Biochemical assessments

Blood samples were collected from all participants after a 12-h overnight fast, centrifuged (2500 rpm, 4 °C, 10 min) and immediately stored at − 80 °C. Analyses to determine total cholesterol, HDL cholesterol, LDL cholesterol, triglycerides, and fasting plasma glucose were performed at a local laboratory. Fasting plasma glucose was determined by the glucose oxidase method; total cholesterol, HDL cholesterol, and triglyceride concentrations were assessed using the enzymatic colorimetric method; and LDL cholesterol was calculated using the Friedewald formula.

Fasting plasma glucose, HDL cholesterol and triglycerides were classified based on 2009 consensus criteria developed by institutions focused on cardiovascular health [22]. Total cholesterol and LDL cholesterol were classified based on the Third Report of the National Cholesterol Education Program [23].

### Blood pressure

Systolic and diastolic blood pressure were measured on both the left and right arms (Omron HEM-705CP®) according to the recommendations of the European Society of Hypertension and the European Society of Cardiology [24]. High blood pressure was diagnosed based on the average of each systolic and diastolic blood pressure measurements from the arm which produced the higher pressure reading [24]. High blood pressure was considered to exist when mean systolic blood pressure was ≥130 mmHg and/or mean diastolic blood pressure was ≥85 mmHg [22]. The intake of medication to treat hypertension was also considered a criterion for determining the presence of risk.

### Dietary assessment

Dietary intake was assessed using a semiquantitative food frequency questionnaire (SQFFQ) that was administered in the interview format and had been validated on a Mexican population [25]. The questionnaire comprised 162 items. Question on *agua fresca* (water with crushed fruits and sugar)   intake was included, as it was commonly consumed in Mexico. Each item presented a standard serving and nine response options ranging from “never” to “more than six servings per day” [26]. On the day of the survey, participants reported their habitual intake frequency and amount of each food over the previous year, and the interviewers (trained nutritionists) selected appropriate response options according to the information provided. Subsequently, the average daily intake of each item was calculated based on the standard serving size and intake frequency of each item and divided by seven if per-week intake frequency was considered. Example: the standard yogurt serving in the SQFFQ is 125 g; if yogurt intake frequency was three servings per week, daily intake was 53.57 g [(125*3)/7]. Finally, the daily intake of the 163 items was grouped into 25 food groups (Table 1) based on the food groupings found in Appendix A of Official Mexican Standard NOM-043-SSA2–2012 (“El Plato del bien comer”, or “plate of good eating” guide) [27]. Items were also grouped according to similarities in the nutritional characteristics of foods (lipid, protein, carbohydrate, fiber or alcohol content) and intake frequency.

**Table 1:** ^a^25 Food groups and the foods that comprise them

Food groups	Foods
1. Milk	Milk and yogurt.
2. Cheese	Cold cheese (cottage cheese and white cheese) and melting cheese.
3. Eggs	Eggs.
4. White meat	Chicken.
5. Red meat	Beef and pork.
6. Processed meats	Ham and sausages: salami, chorizo, longaniza, hot dog.
7. Fish and seafood	Fish, canned tuna in water or oil, and seafood (squid and crustaceans).
8. Animal fats	Butter, cream, bacon, lard, cream cheese.
9. Vegetable fats	Corn, sunflower, soy and olive oils, and avocado.
10. Nuts	Almonds, walnuts and peanuts.
11. Refined cereals	Rice and pasta, white bread and flour tortilla.
12. Whole grains	Whole wheat bread, whole-grain cereals and crackers.
13. Tubers	Potatoes prepared home-style.
14. Autochthonous cereals	Corn tortilla, nixtamal, ear maize and tostada.
15. Legumes	Beans, lentils and chickpeas.
16. Vegetables	Chard, lettuce, cauliflower, broccoli, green beans, chili, celery, cactus, cucumber, zucchini, squash, onion, garlic, mushroom, tomato, carrot and peas.
17. Fruits	Strawberry, plum, grape, dried fruits, bananas, apple, pear, mango, peach, watermelon, cantaloupe, papaya, pineapple, guava, prickly pear, kiwi and citrus.
18. Natural fruit juices	Natural fruit juices.
19. Bakery products and cookies	Sweet industrialized pastries, homemade sweet bread, cake, cookies and breakfast cereals.
20. Sugars	Cajeta, quince cheese, candies, jam, honey, ice cream, chocolate and cocoa powder.
21. Fast food	Pizza, hamburgers and fried potato products.
22. Industrial sauces	Ketchup, hot sauce and mustard.
23. Water	Water.
24. Sweetened drinks	Industrialized juices, soft drinks, naturally sweetened fruit drinks (*aguas frescas*), coffee and tea.
25. Alcoholic beverages	Red wine, beer and distillates.

^a^The 25 food groups were created based on the 163 items in the semiquantitative food frequency questionnaire

[Table 1 should appear here].

### Ethical considerations

The project was carried out according to Declaration of Helsinki guidelines, and all procedures were approved by the Ethics and Research Committee of the University Center for Health Sciences, University of Guadalajara (registration number CI-13909). All participants voluntarily signed an informed consent.

### Statistical analysis

The *Kolmogorov-Smirnov* test was used to assess the normality of quantitative variables. Because the distribution of quantitative variables was asymmetrical, calculations of medians and interquartile ranges were performed. The difference between medians was calculated using either the Mann-Whitney U (for comparisons of two groups) or Kruskal-Wallis (for comparisons of three or more groups) statistical tests. If the result in the Kruskal Wallis test was significant, pairwise comparisons were made using the Mann-Whitney U test adjusted using the Bonferroni method.

Categorical variables were expressed as numbers and percentages. Associations between proportions were calculated using the chi-squared statistical test. 

DPs based on the 25 food groups were generated using the multivariate statistical technique known as the principal components analysis (PCA). Prior to administration of the PCA, Bartlett’s sphericity test (p<0.001) and the Kaiser-Meyer Olkin measurement of sampling adequacy (p=0.717) were administered. These tests determined (criteria: <0.05 y >0.07, respectively, in each test) that the correlation between the variables in question was high enough to make performing a PCA statistically feasible [28, 29]. The number of components was delimited based on the scree plot test (graphic representation of the extracted components versus their eigenvalues) [30]*,* from which we decided to retain three DPs (corresponding to the number of data points above the “break”; i.e., the point where the natural curve flattens out, not including the point at which the break occurs) [30]. The Varimax orthogonal rotation was used to improve the interpretability of the DPs [30] and to obtain unrelated components [30] that subsequently underwent regression analysis. A correlation equal to or greater than 0.3 between the food group and the component was considered significant [30]. Each DP was assigned a name based on the food groups that had the highest load factor. Finally, scores from the DPs were categorized into tertiles for the purposes of description and interpretation, with higher tertile values being associated with greater adherence to the DP.

Associations between DPs and sociodemographic, lifestyle, anthropometric and biochemical variables were analyzed using multivariable logistic regression adjusted for age and sex. For the regression analysis, DP scores were halved into two categories, with the second half representing greater adherence to the DP. P<0.05 was considered significant in all statistical tests. Calculations were performed using SPSS version 18 statistical software for Windows [31].

## Results

### Participant characteristics

A total of 462 subjects were invited to participate in the study; of them, 319 met the inclusion criteria and completed the surveys. The majority of participants were younger than 29 years of age (84%), women (71.2%) and students (59.6%). The most highly represented health fields were nutrition (24.8%) and pharmaceutical biochemistry (22.6%). The majority of participants reported being non-smokers (89.5%) and engaging in more than 150 min of PA per week (98.1%). According to BMI data, 24.5% were overweight, 8.5% were obese and 27.2% presented abdominal obesity (Table 2).

**Table 2:** General characteristics (*n* = 319)

	n	%
Sex		
Men	92	28.8
Women	227	71.2
Age (years)		
18 to 22	162	50.8
23 to 29	106	33.2
≥ 30	51	16.0
Occupational status		
Professional	129	40.4
Student	190	59.6
Health field		
Nutrition	79	24.8
Nursing	50	15.7
Medicine	42	13.2
Psychology	33	10.4
Pharmaceutical biochemistry	72	22.6
Dentistry	37	11.6
^a^Other areas	5	1.6
Smoking status		
Non-smoker	282	89.5
Smoker	33	10.5
^b^BMI		
Underweight	22	6.9
Normal weight	192	60.2
Overweight	78	24.5
Obese	27	8.5
^c^OA		
Yes	86	27.2
No	230	72.8
^d^PA (min/sem)		
< 150	6	1.9
> 150	313	98.1

^a^Other health fields: Biology, Chemistry, Ph.D. in Public Health; ^b^*BMI* Body mass index, ^c^*AO* Abdominal obesity (waist circumference ≥ 80 cm in women and ≥ 90 cm in men (criteria for Central and South American populations)); ^d^*PA* Physical activity

### Description of biochemical and anthropometric parameters

Significantly higher waist circumference, BMI and systolic blood pressure values were found more often in men than in women. Nonetheless, women had higher concentrations of total cholesterol and HDL cholesterol than men (Table 3).

**Table 3:** Description of biochemical and anthropometric parameters among healthcare professionals and students (n = 319)

	Total		Men		Women		^a^P
	Median	25th and 75th percentile	Median	25th and 75th percentile	Median	25th and 75th percentile	
Waist circumference (cm)	77.1	69.5–84.2	82.8	76.9–92.6	74.4	68.0–80.5	0.001*
^b^BMI (kg/m^2^)	23.2	20.8–26.2	24.8	22.0–27.1	22.8	20.5–25.7	0.001*
Diastolic Blood Pressure	69.5	64.2–75.5	71.5	63.7–77.2	68.5	64.5–74.7	0.130
Systolic Blood Pressure	115.0	107.0–124.0	124.0	117.0–132.2	111.0	105.0–118.5	0.001*
Fasting plasma glucose (mmol/L)	72.0	66.5–78.0	72.0	67.5–79.0	72.0	66.0–77.0	0.334
Total Cholesterol (mmol/L)	166.0	142.5–188.5	152.5	133.0–185.0	167.0	146.5–189.0	0.027*
HDL Cholesterol (mmol/L)	52.0	46.0–60.0	49.0	42.0–53.5	55.0	48.0–62.5	0.001*
Triglyceride (mmol/L)	73.0	54.0–106.5	82.0	52.0–130.0	69.0	54.0–101.0	0.103
LDL Cholesterol (mmol/L)	94.0	76.2–114.3	91.3	72.5–114.9	95.1	77.0–114.0	0.388

^a^The difference between medians was calculated using the Mann-Whitney U test. *P < 0.05 was considered significant

^b^*BMI* Body mass index

### Dietary intake

After analyzing the intake of the 25 food groups, the daily intake of water in the majority of the study sample (75%) was found to be less than 1680 ml, while the intake of sweetened beverages exceeded 200 ml per day. In addition, half of the sample consumed at least 200 g of vegetables and at least 300 g of fruits per day (Table 4). Regarding intake by sex, men consumed a significantly (p<0.05) higher quantity of eggs, red meat, processed meat, fish and seafood, nuts, fast food and alcoholic beverages than women (Table 4). Regarding the intake of other food groups, no significant differences between women and men were found (p>0.05) (Table 4).

**Table 4:** Intake of food groups among healthcare professionals and students (n = 319)

Food groups	Total			Men			Women			
	Median	25th and 75th percentile		Median	25th and 75th percentile		Median	25th and 75th percentile		^a^P
Milk (ml/d)	254	148	526	296	143	515	254	155	529	0.504
Cheese (g/d)	24	13	36	24	11	37	23	15	36	0.420
Eggs (g/d)	26	9	26	26	9	47	26	9	26	0.001*
White meat (g/d)	43	14	43	43	14	46	43	14	43	0.134
Red meat (g/d)	56	32	86	65	50	96	51	29	69	0.001*
Processed meat (g/d)	16	8	34	20	13	34	16	8	31	0.040*
Fish and seafood (g/d)	35	21	54	44	28	73	33	20	46	0.001*
Animal fats (g/d)	9	3	15	10	3	15	9	3	15	0.941
Vegetable fats (g/d)	19	12	29	20	12	29	19	12	28	0.708
Nuts (g/d)	7	3	18	11	4	24	6	3	16	0.005*
Refined cereals (g/d)	37	24	56	41	26	61	36	23	53	0.071
Whole-grain cereals (g/d)	22	12	43	20	6	48	24	12	42	0.421
Tubers (g/d)	9	4	16	9	3	26	9	4	16	0.728
Autochthonous cereals (g/d)	78	32	107	82	30	157	72	33	102	0.318
Legumes (g/d)	44	19	68	44	28	71	44	14	65	0.351
Vegetables (g/d)	243	161	332	243	158	339	244	169	328	0.889
Fruit (g/d)	306	193	431	289	170	421	310	200	444	0.238
Natural fruit juices (ml/d)	27	0	86	27	0	86	13	0	57	0.299
Bakery products and cookies (g/d)	42	26	67	47	27	79	38	25	65	0.128
Sugars (g/d)	26	14	47	26	13	53	26	14	44	0.932
Fast food (g/d)	29	17	49	37	19	78	24	17	41	0.005*
Industrial sauces (g/d)	6	3	10	7	4	12	6	3	10	0.099
Water (ml/d)	1200	600	1680	1200	600	1680	1200	600	1680	0.309
Sweetened drinks (ml/d)	347	205	686	437	209	772	330	189	680	0.148
Alcoholic beverages (ml/d)	29	3	141	54	22	191	25	3	61	0.001*

^a^The difference between medians was calculated using the Mann-Whitney U test. *P < 0.05 was considered significant

### Description of dietary patterns

Three DPs were retained and collectively accounted for 29.28% of the total variance. All food groups selected for each pattern (highlighted in bold in Table 5) showed significant correlations with the component (r= ≥0.3), with the exception of the natural fruit juices group (r=0.227). This group remained in the PCA due to its importance in the makeup of the DP.

**Table 5:** ^a^Dietary patterns of Mexican health students and professionals (n = 319)

Food groups	Traditional Westernized	Healthy	Animal protein and Alcoholic beverages
Bakery products and cookies	**0.637**	- 0.160	0.204
Sugars	**0.594**	0.088	0.184
Refined cereals	**0.537**	0.109	0.357
Fast food	**0.483**	0.046	0.416
Tubers	**0.470**	0.284	0.060
Legumes	**0.446**	0.100	- 0.185
Autochthonous cereals	**0.413**	0.047	- 0.057
Sweetened drinks	**0.409**	0.002	0.144
Animal fats	**0.387**	0.138	0.075
Milk	**0.349**	- 0.031	0.001
Vegetables	0.189	**0.752**	- 0.161
Fruits	0.203	**0.660**	- 0.154
Whole grains	0.147	**0.511**	- 0.088
Water	- 0.254	**0.438**	0.000
Nuts	- 0.123	**0.433**	0.098
White meat	- 0.146	**0.406**	0.345
Fish and seafood	- 0.023	**0.344**	0.335
Cheese	0.141	**0.326**	0.131
Vegetable fats	0.205	**0.310**	- 0.018
Natural fruit juices	0.051	**0.227**	0.186
Red meat	0.130	- 0.084	**0.631**
Eggs	- 0.006	0.007	**0.590**
Processed meats	0.230	0.121	**0.562**
Alcoholic beverages	- 0.011	- 0.049	**0.534**
Industrial sauces	0.236	0.015	**0.338**
Variance (%)	14.18	8.78	6.32

^a^Dietary patterns were generated using principal component analyses

The first component accounted for 14.18% of the total variance and was categorized as “Traditional Westernized” because it consisted mainly of traditional Mexican foods such as tubers, legumes, autochthonous cereals, animal fats and milk, as well as foods common in Western culture such as bakery products and cookies, sugars, refined cereals, fast food and sweetened beverages (Table 5). The second DP accounted for 8.78% of the variance and was classified as “Healthy” because it contained vegetables, fruits, whole grains, water, nuts, white meats, fish and seafood, cheese, vegetable fats and natural fruit juices (Table 5). Finally, the third DP (accounting for 6.32% of the variance) was named “Animal protein and alcoholic beverages” due to the presence of animal protein sources such as red meat, eggs, processed meat, alcoholic beverages and industrial sauces (Table 5).

Participants in the highest “Traditional Westernized” DP adherence tertile were more frequently categorized (p<0.05) as being under the age of 22 years (37.7%), students (38.9%) and active in the nursing (50%) or psychology (60.6%) fields. Subjects most frequently found (p<0.05) in the third adherence tertile of the “Healthy” DP were individuals over the age of 30 (54.9%), professionals (43.4%), active in the medical field (57.1%), overweight (42.3%) or obese (40.7%), and subjects whose daily PA energy expenditure was greater than 605 kcal (49.1%). Male participants (50%) and those who reported being smokers (60.6%) appeared more frequently (p<0.05) in the highest adherence tertile of the “Animal protein and alcoholic beverages” DP (Table 6).

**Table 6:** Description of dietary patterns according to sociodemographic, lifestyle and anthropometric characteristics (n = 319)

		Traditional Westernized				Healthy				Animal protein & alcoholic beverages			
	Total	*n* = 107	*n* = 105	n = 107	^a^P	*n* = 106	n = 107	n = 106	^a^P	n = 107	n = 106	n = 106	^a^P
		^b^T1(%)	T2(%)	T3(%)		T1(%)	T2(%)	T3(%)		T1(%)	T2(%)	T3(%)	
Sex													
Men	92	34.8	26.1	39.1	0.194	32.6	28.3	39.1	0.309	19.6	30.4	50.0	0.001*
Women	227	32.6	36.1	31.3		33.9	35.2	30.8		39.2	34.4	26.4	
Age (years)													
18 to 22	162	25.3	37.0	37.7	0.004*	38.3	33.3	28.4	0.006*	32.7	30.2	37.0	0.597
23 to 29	106	34.9	32.1	33.0		31.1	38.7	30.2		33.0	37.7	29.2	
≥ 30	51	54.9	23.5	21.6		23.5	21.6	54.9		37.3	33.3	29.4	
Health field													
Nutrition	79	43.0	38.0	19.0	0.001*	24.1	39.2	36.7	0.007*	44.3	34.2	21.5	0.247
Nursing	50	18.0	32.0	50.0		40.0	36.0	24.0		28.0	36.0	36.0	
Medicine	42	47.6	19.0	33.3		26.2	16.7	57.1		28.6	35.7	35.7	
Psychology	33	18.2	21.2	60.6		24.2	45.5	30.3		36.4	24.2	39.4	
Pharmaceutical biochemistry	72	34.7	43.1	22.2		43.1	34.7	22.2		30.6	37.5	31.9	
Dentistry	37	32.4	29.7	37.8		40.5	27.0	32.4		24.3	24.3	51.4	
Other areas	5	0.0	60.0	40.0		40.0	0.0	60.0		40.0	40.0	20.0	
Occupational status													
Professional	129	43.4	31.0	25.6	0.004*	27.9	28.7	43.4	0.006*	34.1	35.7	30.2	0.609
Student	190	26.3	34.7	38.9		37.4	36.3	26.3		33.2	31.6	35.3	
^d^BMI													
Underweight	22	13.6	45.5	40.9	0.350	59.1	31.8	9.1	0.030*	40.9	27.3	31.8	0.394
Normal weight	192	33.3	32.8	33.9		33.9	34.9	31.3		31.8	38.0	30.2	
Overweight	78	41.0	29.5	29.5		24.4	33.3	42.3		33.3	25.6	41.0	
Obese	27	25.9	37.0	37.0		37.0	22.2	40.7		40.7	25.9	33.3	
^e^AO					0.266				0.179				0.787
Yes	86	27.9	31.4	40.7		25.6	34.9	39.5		36.0	30.2	33.7	
No	230	34.8	33.9	31.3		36.1	32.6	31.3		32.6	33.9	33.5	
Smoking status					0.722				0.737				0.002*
Non-smoker	282	33.3	33.3	33.3		33.7	32.6	33.7		35.5	34.0	30.5	
Smoker	33	27.3	33.3	39.4		30.3	39.4	30.3		15.2	24.2	60.6	
^f^PA energy expenditure (kcal/day)													
≤ 345.0	107	35.5	33.6	30.8	0.948	47.7	32.7	19.6	0.001*	34.6	36.4	29.0	0.648
345.1 to 605.2	106	33.0	33.0	34.0		33.0	35.8	31.1		31.1	34.9	34.0	
≥ 605.3	106	31.1	33.0	35.8		19.8	33.1	49.1		34.9	28.3	36.8	

^a^The association between proportions was calculated using the chi-squared statistical test. *P < 0.05 was considered significant

^b^T: tertile

^c^Other health fields: Biology, Chemistry, Ph.D. in Public Health

^d^BMI: Body mass index

^e^AO: Abdominal obesity (waist circumference ≥ 80 cm in women and ≥ 90 cm in men (criteria for Central and South American populations))

^f^PA: Physical activity

Waist circumference and BMI were significantly higher among subjects classified in the third “Healthy” DP adherence tertile than among those in the first tertile (*p* < 0.05). Systolic blood pressure was also significantly higher among those in the third “Animal protein and alcoholic beverages” DP adherence tertile than among those others in the first tertile (*p* = 0.05). No significant differences between tertiles of each of the three DPs were found for the remaining biochemical and anthropometric parameters (Table 7).

**Table 7:** Description of dietary patterns according to anthropometric and biochemical parameters (n = 319)

	^a^T1		T2		T3		
	Median	25th and 75th percentile	Median	25th and 75th percentile	Median	25th and 75th percentile	^b^P
	Traditional Westernized Dietary Pattern						
Waist circumference (cm)	77.4	70.4–84.6	76.0	68.9–82.6	78.6	69.0–86.5	0.561
^c^BMI (kg/m^2^)	24.1	21.4–26.6	23.2	20.5–26.0	23.0	20.1–26.2	0.201
Diastolic Blood Pressure (mm Hg)	69.2	64.5–75.0	69.0	63.5–76.5	69.5	64.0–75.0	0.995
Systolic Blood Pressure (mm Hg)	117.0	107.2–123.2	115.0	107.5–125.0	115.0	106.0–124.0	0.871
Fasting plasma glucose (mg/dL)	72.0	66.5–77.0	71.0	66.0–76.0	72.0	67.0–79.0	0.373
Total Cholesterol (mg/dL)	166.5	138.5–196.0	169.0	145.0–193.0	163.0	143.0–179.0	0.323
HDL Cholesterol (mg/dL)	52.0	46.0–59.5	54.0	47.0–61.0	52.0	46.0–59.0	0.590
Triglyceride (mg/dL)	72.0	52.5–104.5	73.0	54.0–104.0	75.0	55.0–113.0	0.831
LDL Cholesterol (mg/dL)	95.7	73.0–119.2	95.0	78.0–119.5	92.0	76.4–107.0	0.670
	Healthy Dietary Pattern						
Waist circumference (cm)	74.2	67.3–82.3	78.0	71.3–82.5	78.7	70.4–88.7	0.015*
^c^BMI (kg/m^2^)	22.3	20.2–25.3	23.1	20.9–25.8	24.3	21.8–27.3	0.003*
Diastolic Blood Pressure (mm Hg)	70.5	65.5–75.5	69.0	64.5–73.5	69.5	63.5–76.5	0.470
Systolic Blood Pressure (mm Hg)	115.0	107.5–123.0	115.0	106.0–124.0	117.0	107.0–124.5	0.698
Fasting plasma glucose (mg/dL)	71.0	66.0–78.0	71.0	66.0–78.0	72.5	67.0–78.0	0.349
Total Cholesterol (mg/dL)	164.0	141.0–186.0	164.0	142.0–183.0	172.5	148.0–199.0	0.057
HDL Cholesterol (mg/dL)	51.0	47.0–61.0	52.0	46.0–60.0	54.0	46.0–60.0	0.705
Triglyceride (mg/dL)	75.0	55.0–107.0	67.0	49.0–101.0	77.5	57.0–110.0	0.200
LDL Cholesterol (mg/dL)	91.0	72.0–111.8	92.4	77.0–107.0	98.0	76.8–123.2	0.115
	Animal protein & alcoholic beverages Dietary Pattern						
Waist circumference (cm)	75.8	68.0–82.5	75.9	69.2–83.0	79.1	72.1–86.1	0.061
^c^BMI (kg/m^2^)	23.2	20.5–26.3	23.0	21.0–25.5	23.6	21.1–26.8	0.698
Diastolic Blood Pressure (mm Hg)	69.0	64.5–75.0	70.2	64.5–75.2	69.2	63.5–75.5	0.956
Systolic Blood Pressure (mm Hg)	112.0	105.5–120.0	117.0	108.7–123.5	117.0	108.0–126.0	0.041*
Fasting plasma glucose (mg/dL)	71.0	67.0–75.0	72.0	66.0–79.0	72.0	66.0–78.0	0.399
Total Cholesterol (mg/dL)	165.0	142.0–181.0	166.5	143.0–192.5	167.0	142.0–191.0	0.644
HDL Cholesterol (mg/dL)	52.5	46.0–60.0	52.0	48.0–59.0	52.5	45.0–61.0	0.969
Triglyceride (mg/dL)	65.5	53.0–102.0	76.5	53.0–102.5	79.5	57.0–119.0	0.232
LDL Cholesterol (mg/dL)	88.7	74.0–106.8	98.0	78.4–115.2	92.2	77.0–119.2	0.298

^a^T: tertile

^b^The difference between medians was calculated using the Kruskal Wallis. *P < 0.05 was considered significant

^c^BMI: Body Mass Index

After adjusting for sex, being younger than 22 years was positively associated with the “Traditional Westernized” DP and negatively associated with the “Healthy” DP. After adjusting for age and sex, the “Healthy” DP was also negatively associated with being a student and positively associated with having a daily PA energy expenditure greater than 605 kcal. Finally, being male and smoking were positively associated with the “Animal protein and alcoholic beverages” DP, after adjustment. No significant associations were found between the DPs and the anthropometric and biochemical variables; that is, these variables do not seem to favor adherence to DPs (Table 8).

**Table 8:** ^a^Association between dietary patterns and sociodemographic, lifestyle and anthropometric characteristics (n = 319)

		Traditional Westernized		Healthy		Animal protein & alcoholic beverages	
	n	Crude OR CI (95%)	Adjusted OR CI (95%)^b^	Crude OR CI (95%)	Adjusted OR CI (95%)^b^	Crude OR CI (95%)	Adjusted OR CI (95%)^b^
Sex							
Women	227	1.00	1.00	1.00	1.00	1.00	1.00
Men	92	1.14 (0.70, 1.85)	1.22 (0.74, 1.99)	1.19 (0.73, 1.93)	1.13 (0.69, 1.84)	2.91 (1.74, 4.86)*	3.05 (1.81, 5.14)*
Age (years)							
18 to 22	162	2.13 (1.11, 4.08)*	2.16 (1.12, 4.15)*	0.44 (0.23, 0.84)*	0.44 (0.23, 0.85)*	1.53 (0.81, 2.88)	1.72 (0.89, 3.34)
23 to 29	106	1.90 (0.96, 3.79)	1.93 (0.97, 3.85)	0.59 (0.29, 1.17)	0.59 (0.29, 1.18)	1.22 (0.62, 2.39)	1.34 (0.67, 2.70)
≥ 30	51	1.00	1.00	1.00	1.00	1.00	1.00
Occupational status							
Professional	129	1.00	1.00	1.00	1.00	1.00	1.00
Student	190	1.72 (1.09, 2.69)*	1.39 (0.81, 2.37)	0.49 (0.31, 0.78)*	0.55 (0.32, 0.94)*	1.49 (0.95, 2.34)	1.38 (0.80, 2.39)
^c^BMI							
Underweight	22	2.09 (0.82, 5.38)	2.01 (0.78, 5.16)	0.40 (0.15, 1.06)	0.42 (0.16, 1.12)	0.82 (0.34, 1.98)	0.83 (0.34, 2.07)
Normal weight	192	1.00	1.00	1.00	1.00	1.00	1.00
Overweight	78	0.72 (0.42, 1.22)	0.77 (0.45, 1.35)	1.70 (0.99, 2.91)	1.53 (0.88, 2.66)	1.14 (0.67, 1.94)	1.03 (0.59, 1.81)
Obese	27	1.05 (0.47, 2.36)	1.25 (0.54, 2.90)	0.98 (0.44, 2.21)	0.82 (0.35, 1.90)	0.67 (0.30, 1.53)	0.60 (0.25, 1.44)
Abdominal Obesity							
No	230	1.00	1.00	1.00	1.00	1.00	1.00
Yes	86	1.12 (0.68, 1.83)	1.38 (0.81, 2.35)	1.35 (0.82, 2.23)	1.17 (0.69, 1.97)	1.00 (0.61, 1.64)	1.09 (0.64, 1.87)
Smoking status							
Non-smoker	282	1.00	1.00	1.00	1.00	1.00	1.00
Smoker	33	1.05 (0.51, 2.15)	1.03 (0.50, 2.14)	0.71 (0.34, 1.46)	0.68 (0.33, 1.43)	2.94 (1.32, 6.56)*	2.69 (1.18, 6.12)*
^d^PA energy expenditure (kcal/day)							
≤ 345.0	107	1.00	1.00	1.00	1.00	1.00	1.00
345.1 to 605.2	106	1.06 (0.62, 1.81)	0.99 (0.57, 1.71)	1.68 (0.97, 2.92)	1.88 (1.07, 3.31)*	1.23 (0.72, 2.11)	1.23 (0.70, 2.15)
≥ 605.3	106	0.88 (0.51, 1.50)	0.80 (0.46, 1.38)	3.68 (2.09, 6.49)*	4.17 (2.32, 7.49)*	1.10 (0.64, 1.88)	0.97 (0.55, 1.70)
High Blood Pressure							
No	264	1.00	1.00	1.00	1.00	1.00	1.00
Yes	52	0.68 (0.37, 1.24)	0.72 (0.37, 1.38)	0.98 (0.54, 1.78)	0.78 (0.41, 1.50)	1.45 (0.79, 2.64)	1.05 (0.54, 2.10)
High HDL-Cholesterol							
No	232	1.00	1.00	1.00	1.00	1.00	1.00
Yes	84	0.66 (0.40, 1.10)	0.67 (0.40, 1.11)	0.76 (0.46, 1.25)	0.77 (0.46, 1.27)	0.77 (0.47, 1.27)	0.88 (0.53, 1.48)
Hipertriglyceridemia							
No	280	1.00	1.00	1.00	1.00	1.00	1.00
Yes	36	1.12 (0.56, 2.24)	1.38 (0.66, 2.90)	1.12 (0.56, 2.24)	0.89 (0.43, 1.86)	0.88 (0.44, 1.77)	0.73 (0.34, 1.55)
High LDL-Cholesterol							
No	185	1.00	1.00	1.00	1.00	1.00	1.00
Yes	131	0.86 (0.55, 1.34)	0.97 (0.61, 1.54)	1.45 (0.92, 2.27)	1.35 (0.85, 2.15)	1.14 (0.73, 1.78)	1.36 (0.84, 2.18)
Hypercholesterolemia							
No	257	1.00	1.00	1.00	1.00	1.00	1.00
Yes	59	0.73 (0.42, 1.30)	0.99 (0.53, 1.85)	1.03 (0.58, 1.81)	0.75 (0.40, 1.41)	0.96 (0.54, 1.69)	1.16 (0.61, 2.20)

^a^The association between dietary patterns and sociodemographic, lifestyle, anthropometric and biochemical variables was analyzed using logistic regression. Dietary patterns scores were halved into two categories, with the second half representing greater adherence to the pattern. The results are expressed as Odds Ratio (OR) with 95% Confidence interval (CI)

^b^Adjusted for age and sex (sex is presented adjusted for age and age is presented adjusted for sex). *P < 0.05 was considered significant

^c^BMI: Body mass index. ^d^PA: Physical activity

## Discussion

Among the healthcare students and professionals assessed in this study, three DPs were identified: “Traditional Westernized”, “Healthy” and “Animal protein and alcoholic beverages”. After adjustment, the “Traditional Westernized” DP was positively associated with being younger than 22 years; the “Healthy” DP was positively associated with a daily PA energy expenditure greater than 605 kcal and negatively associated with being younger than 22 years; and the “Animal protein and alcoholic beverages” DP was positively associated with being both a man and a smoker.

The “Traditional Westernized” DP presented the highest percentage of variance. A DP that includes a combination of traditional and Western-style foods had already been reported in the Mexican population [32–34]. Participants in the baseline assessment of the Health Workers Cohort Study (employees and their relatives from three different health and academic institutions from Mexico) showed a DP that contained corn *tortilla* (a traditional Mexican food) as well as pastries, refined cereals and soft drinks [32]. In another cross-sectional study, a sample of adult women from Tijuana, a Mexican city at the Mexico-United States border, presented a DP containing Mexican *burritos* and sweets (traditional foods from northern Mexico), as well as hamburgers and pizza, snack foods, sweetened drinks, bakery products and peanut butter [33]. Furthermore, a DP comprised of Mexican food and *tortilla* in combination with refined grains, desserts, sweets and sugar, snacks, sweet beverages, high-fat dairy, among others, was observed in Mexican adolescents (14–16 years old) from the State of México, in a cross-sectional study [34]. No studies were found which evaluated the DPs of only health students or health professionals from Mexico or any other country.

One of the possible reasons for the presence of a DP that included both traditional Mexican and Western-style foods is that Mexico is undergoing a nutritional transition fueled by its urbanization and socioeconomic growth trends [1, 2, 35]. Although corn and beans are still found in the Mexican DP, the proportion of energy intake from these foods has decreased in the last 50 years (corn intake has dropped from 46.2 to 34%, and beans from 5.8 to 3.4%) [36]. At the same time, increases have been reported in the proportion of energy obtained from sugar (from 12.6 to 15.4%), vegetable oils (from 5.6 to 8.2%), butter (from 0.28 to 0.41%), poultry (from 0.64 to 2.4%), eggs (from 0.68 to 1.6%) and animal fats (from 1.1 to 2.2%) [36]. The Traditional DP is undergoing a shift towards a more Western-style DP [35, 37] characterized by energy-dense foods containing high levels of total and saturated fats, refined carbohydrates and salt. The higher intake levels of these foods are attributable to their increased availability, sales, and consumption [1, 2, 35, 37, 38]. In Mexico, many foods with these characteristics can be more easily acquired and consumed due to an increase in the number of establishments that sell them, including conventional restaurants, small family-operated restaurants, small grocery stores, supermarkets and convenience stores [35, 37]. These establishments have benefited from a reduction in the time that people have to cook and from increased advertising of food and beverage products [2].

This study also found a positive association between the “Traditional Westernized” DP and being under the age of 22 years. A similar result was obtained in a cross-sectional study of women from a Mexican city: young adults (18 to 29 years) and students received the highest scores on a DP that included both traditional and Western-style foods [33]. The fact that the young adults in this study (who are mainly health university students) show greater adherence to a DP that includes bakery products, cookies, sugars, refined cereals, fast food, and sweetened beverages may be attributable to the main barriers to following a healthy diet that were reported by university students in a review study [39]. These barriers include the following: preference for the taste of unhealthy foods; lack of self-regulation of behavior and lack of motivation to eat a healthy diet; lack of skills and time needed to plan, buy and prepare or cook healthy foods; increased appetites and a preference for unhealthy foods as a response to emotional states such as stress; unhealthy dietary influences of friends and family; the lower cost of unhealthy foods; and the widespread presence of unhealthy foods in restaurants, dining halls, etc. [39]. These barriers may also explain the negative association found between the “Healthy” PD and being a student.

The second DP identified in this study was “Healthy”. A DP similar to this one has already been observed in other cross-sectional studies on Mexican adults (18 to 70 years old) [32, 33], and among young study participants from Mexico (14 to 16 years old) [34], Brazil (average age of 23 years) [40] and the US (19 to 39 years old) [41, 42] in categories such as “Prudent” [32, 34, 41, 42], “Fruits and vegetables” [40] and “Vegetables” [33]. In this study, the “Healthy” DP was negatively associated with being younger than 22 years and positively with having a daily PA energy expenditure of more than 605 kcal. In the studies mentioned above that have observed this DP, it has also been found that those who more frequently adhere to a DP comprised of healthy foods are older [32, 41], less sedentary [32] and more likely to engage in PA than other subjects [40]. They also tend to be women [40], to have completed more years of education [33, 41], to present overweight or obesity less frequently [41] and to smoke less [32] or be non-smokers [34, 40, 41]. Despite the absence of an association between DPs and anthropometric variables in this study, waist circumference and BMI were found to be significantly higher among subjects in the third “Healthy” DP adherence tertile than among those in the first tertile. This finding may be attributable to subjects with obesity wanting to improve their diet and lifestyle in order to lose weight or reduce their waist circumference. This result may also be attributable to an over-reporting of healthy foods. Over-reporting low energy intake has been suggested as being more common in overweight individuals than in those with a healthy weight. This phenomenon may also be associated with a body image disorder or, particularly in this case, a desire to meet the social expectations imposed on health professionals [43].

The third DP observed in this study was named “Animal protein and alcoholic beverages”. This DP generally associated with high meat, egg and alcoholic beverage intake was previously observed in another cross-sectional study that evaluated Mexican adults (20 to 70 years old) [32] under the name of “High in protein and fat” [32]. In line with the findings of this study, individuals who followed a DP with these characteristics were more likely to be men [32] and smokers [32]. It has also been shown that DPs with high levels of alcohol intake tend to be followed in higher percentages by men and smokers [18].

Although the “Healthy” DP was observed among young healthcare professionals assessed in this study, it was negatively associated with higher waist circumference, body fat [42] and BMI [34, 42] measurements in other studies. The “Traditional Westernized” and the “Animal protein and alcoholic beverages” DPs were also observed in this population. Another study on a Mexican population has already positively associated the latter two DPs with metabolic syndrome and its components [32]. The presence of DPs considered unhealthy among these healthcare students and professionals may be explained by the Ecological Model of Health Behavior [44, 45]. This model states that while food selection (health behavior) may be positively or negatively influenced by intrapersonal determinants such as health education, attitudes, and prevention skills, it may also be influenced by the combined actions of interpersonal, organizational, community determinants and public policy. That is, as in the case of the general population, getting healthcare professionals to follow a “Healthy” DP is a question of providing education and ensuring that the attitudes, values, norms, beliefs and behaviors of their social groups (family, work groups, friends), social institutions (schools, workplaces, churches, professional groups) and the communities to which they belong –in addition to political and business leaders – will encourage individual efforts to engage in healthy behaviors [44, 45]. However, study data show that a range of organizational barriers (long work schedules, shift work, heavy workloads, insufficient staff and short, infrequent breaks), social barriers (eating habits of peers), physical workplace barriers (limited access to healthy foods in dining halls or vending machines, inadequate food preparation and conservation facilities, the lower cost and higher availability of junk food compared to healthy foods) and individual barriers (lack of self-efficacy and motivation, and inadequate knowledge about nutrition) make it more difficult for health professionals to engage in healthy eating behaviors [46]. These barriers could account for the presence of the “Traditional Westernized” and “Animal protein and alcoholic beverages” DPs in this study sample.

Considering the presence of unhealthy DPs, it is clear that the healthcare students and health professionals who were assessed, and particularly those of a young age, require intervention initiatives aimed at promoting adherence to a healthy DP that will directly benefit their own health and indirectly benefit the health of the general population under their care. To achieve sustainable changes in behavior that are in line with the Ecological Model of Health Behavior, public policies must be complemented by interventions at the individual, social and organizational [46] levels. Furthermore, the impact of such actions must be assessed to prevent the misuse of resources. Considering the main dietary problems of this evaluated population group and based on the main barriers that university students [39] and health professionals [46] report as preventing them from following a healthy diet, the following measures (in conjunction with existing Mexican public policies such as taxes on sweetened industrialized beverages and junk food, and stepped up regulations on the marketing and labeling of these foods) are suggested in order to promote adherence to a healthy DP within this population: promoting the availability, affordability and daily intake of drinking water, vegetables, fruits, and other healthy food choices at school, workplaces and public venues; developing or improving workplace areas in which food is prepared and stored.

The results presented in this study should be interpreted with consideration for their limitations. Stratified random sampling by age and sex was attempted at the beginning of the study, but it was subsequently changed to volunteer sampling due to unwillingness on the part of the selected population to participate. Consequently, our results here cannot be generalized to the universe of study. However, considering the limited scientific evidence available on the description of the DPs of healthcare personnel, this analysis serves as a starting point to know the food combinations routinely consumed by this population group. Another limitation is the fact that performing the PCA could lead researchers to make subjective decisions regarding how diets should be assessed, how food groupings should be created, the number of components that should be retained, rotation modes and assigning names to DPs [16, 18, 47]. Nonetheless, the PCA is a statistical method that is widely used to generate DPs for published scientific studies [18, 32, 40–42], and DP descriptions may represent habitual food intake and availability more accurately than assessments of individual foods and nutrients within a study population. Furthermore, the DPs observed in this group population were similar to those presented in other studies that evaluated Mexican populations. In addition, and in order to minimize the limitations of the PCA, this study provides detailed descriptions about how decisions were made as well as their theoretical bases. Finally, diets were evaluated by trained nutritionists using a validated SQFFQ which, despite being subject to memory bias and the under- or over-reporting of food intake, offers advantages for the assessment of habitual diet (it enables food intake to be estimated over relatively long periods and for the effects of daily food choice variations to be minimized) compared to other evaluation techniques [48]. Furthermore, an adjustment was made to account for the seasonality of the foods surveyed.

## Conclusions

Among the healthcare students and professionals assessed in this study, three DPs were observed: “Traditional Westernized”, “Healthy” and “Animal protein and alcoholic beverages”; of these, the first and the third DPs were considered unhealthy. After adjustment, the “Traditional Westernized” DP was positively associated with being younger than 22 years; the “Healthy” DP was positively associated with a high daily PA energy expenditure (greater than 605 kcal) and negatively associated with being under 22 years of age and a student; and the “Animal protein and alcoholic beverages” DP was positively associated with being both a man and a smoker. No DP was associated with the analyzed anthropometric and biochemical variables. These data suggest that receiving training in health fields does not ensure that individuals will engage in healthy behaviors. As in the case of the general population, educational and self-help efforts aimed at fostering healthy behaviors (as defined by the Ecological Model of Health Behavior) among healthcare professionals must be reinforced through initiatives undertaken by social groups, social institutions, the community at large as well as political and business leaders. Intervention actions in this evaluated population group designed to promote adherence to a “Healthy” DP must be implemented at the individual, social, organizational and public policy levels. Such initiatives should be particularly suitable for the youngest members of this population and focused on helping them to achieve a low-risk health status and thus indirectly benefit the health of the general population under their care.

## Abbreviations

BMI: Body Mass Index
DPs: dietary patterns
LATINMETS: Latin America Metabolic Syndrome
PA: physical activity
PCA: principal components analysis
SQFFQ: Semiquantitative food frequency questionnaire
